# Computational methods for analysis and inference of kinase/inhibitor relationships

**DOI:** 10.3389/fgene.2014.00196

**Published:** 2014-06-30

**Authors:** Fabrizio Ferrè, Antonio Palmeri, Manuela Helmer-Citterich

**Affiliations:** Centre for Molecular Bioinformatics, Department of Biology, University of Rome Tor VergataRome, Italy

**Keywords:** kinase inhibitors, kinase activity modulation, kinase/inhibitor inference, drug design and development, chemogenomics

## Abstract

The central role of kinases in virtually all signal transduction networks is the driving motivation for the development of compounds modulating their activity. ATP-mimetic inhibitors are essential tools for elucidating signaling pathways and are emerging as promising therapeutic agents. However, off-target ligand binding and complex and sometimes unexpected kinase/inhibitor relationships can occur for seemingly unrelated kinases, stressing that computational approaches are needed for learning the interaction determinants and for the inference of the effect of small compounds on a given kinase. Recently published high-throughput profiling studies assessed the effects of thousands of small compound inhibitors, covering a substantial portion of the kinome. This wealth of data paved the road for computational resources and methods that can offer a major contribution in understanding the reasons of the inhibition, helping in the rational design of more specific molecules, in the *in silico* prediction of inhibition for those neglected kinases for which no systematic analysis has been carried yet, in the selection of novel inhibitors with desired selectivity, and offering novel avenues of personalized therapies.

## INTRODUCTION

**FIGURE 1:**
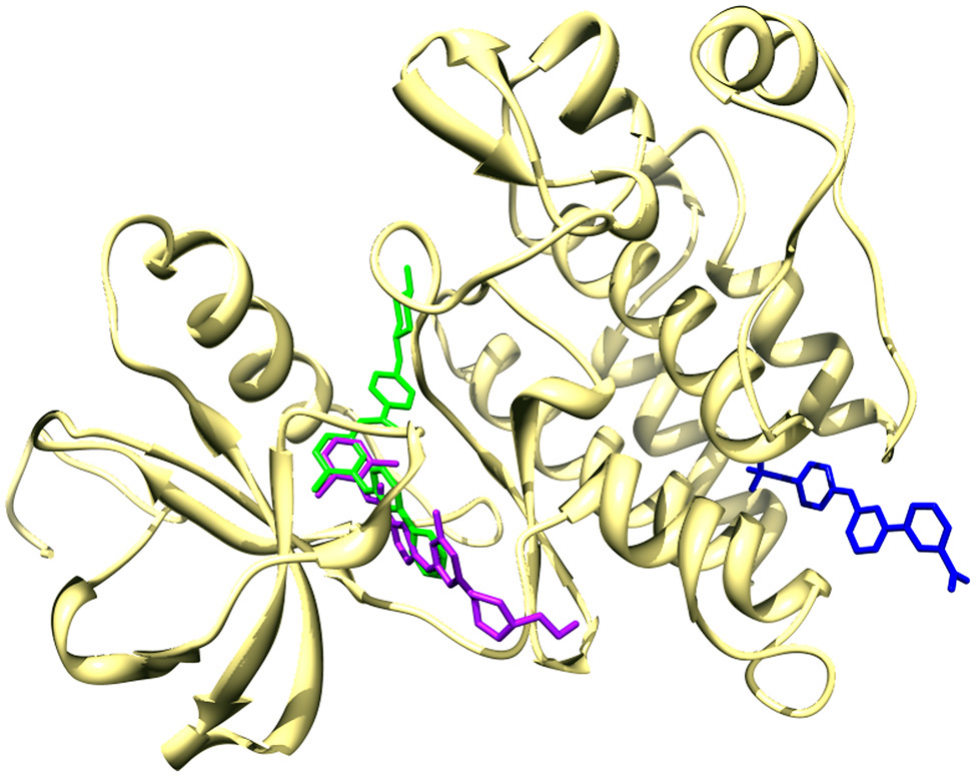
**Binding of the ABL kinase with *dasatinib* (type I inhibitor, shown in purple), *imatinib* (type II inhibitor, shown in green), and *GNF-2* (type IV inhibitor, shown in blue).** The human ABL kinase co-crystallized with *imatinib* (PDB code 1IEP) was used as reference for the structural superposition of the human ABL co-crystallized with *dasatinib* (PDB code 2GQG) and of the mouse ABL in complex with the allosteric inhibitor *GNF-2* (PDB code 3K5V). Only the ribbon representation of the human ABL kinase domain from 1IEP (chain A) is shown.

The kinome plays a predominant role in signal transduction networks and cellular responses; its involvement in a large number of pathologies is a major impulse for the identification and development of compounds modulating the activity of individual kinases or kinase families. Currently, eleven kinase inhibitors are FDA-approved for cancer treatment, and 149 inhibitors and 42 distinct kinase targets are being tested in clinical trials (Fedorov et al., 2010; Chahrour et al., 2012; see http://www.brimr.org/PKI/PKIs.htm for an updated list). In addition to their promises as therapeutical agents, kinase inhibitors are commonly used as research tools to disclose the biological consequences of the inactivation of their targets. Generally, kinase inhibitors are ATP-mimetic compounds. The majority of known inhibitors belong to the so-called type I class, and they occupy directly the ATP binding site, located in a hydrophobic cleft between the two lobes of the kinase domain, while type II inhibitors target the ATP binding site as well, but extend also to an allosteric pocket adjacent to the ATP binding site; additional non-ATP-mimetic inhibitor classes (type III, IV, and V), of which a limited number of examples is currently known, seem very promising therapeutic agents given their generally high specificity (Liu and Gray, 2006; Garuti et al., 2010; Chahrour et al., 2012; Gavrin and Saiah, 2013). An example of type I, II, and IV inhibitors is provided in **Figure 1**. For type I and II inhibitors, the evolutionary structural conservation of the kinase ATP-binding site can lead to off-target binding, and while similar kinases tend to show similar inhibition profiles by sharing recurring sequence and structural patterns (Chiu et al., 2013), often complex kinase/inhibitor relationships occur, where kinase bioactivity profiles cannot be reconciled to their phylogenetic relationships (Paricharak et al., 2013). While absolute specificity toward an individual kinase is not always necessary for a compound to achieve a therapeutic effect (Mencher and Wang, 2005), a detailed knowledge of target selectivity for kinase inhibitors is crucial for predicting and interpreting the effects of inhibitors, and for designing drugs with a desired selectivity. However, kinase inhibitor selectivity is generally not inclusively known for the majority of the tested compounds, as kinase research has been principally focused on a small subset of the kinome.

Traditional kinase inhibitor analysis is a low-throughput process in which the capability of small compounds to decrease the phosphorylation activity (usually reported as the IC_50_ or as the remaining or residual activity of the kinase) or their binding affinity (as its dissociation constant) is measured, but are generally not extended to the characterization of the inhibitory abilities of a given compound against the entire kinome. Such data are mined from the literature and collected in general-purpose databases such as ChEMBL (Gaulton et al., 2012) and STITCH (Kuhn et al., 2014), or in kinase-dedicated public resources such as the CheEMBL Kinase SARfari, or the commercially available Kinase Knowledgebase (KKB) by Eidogen-Sertanty (Oceanside, CA, USA) and the kinase inhibitor database provided by GVK Biosciences (Hyderabad, India). While largely populated, such databases tend to be highly heterogeneous by including evidences obtained by diverse means.

However, in recent years the results of medium- and high-throughput profiling studies became available, tackling inhibition of the phosphorylation activity for panels of widely used research compounds and clinical agents against large subsets of the human kinome (**Table 1**). These studies were able to identify novel inhibitor chemotypes for specific kinase targets and to reveal the target specificities of a large set of kinase inhibitors. Importantly, these panels also provide negative results, i.e., inhibitors having little or no effect on tested kinases, which are instrumental for computational learning techniques and are generally absent or scarce in low-throughput settings.

**Table 1:** Kinase/inhibitor profiling panels.

Dataset	Kinases	Compounds	Readout
Fabian et al. (2005)	119	20	K_d_
Bain et al. (2007)	70–80*	65	% Activity; IC_50_
Fedorov et al. (2007)	60	156	**Δ**T_m_
Bamborough et al. (2008)	203	577	% Control
Karaman et al. (2008)	317	38	K_d_
Posy et al. (2011)	317–402*	21,851	% Control
Davis et al. (2011)	442	72	K_d_
Miduturu et al. (2011)	353	118	% Control; K_d_
Metz et al. (2011)	172	3,858	K_i_
Anastassiadis et al. (2011)	300	178	% Activity
Cao et al. (2013)	234	158	% Activity; IC_50_
Sutherland et al. (2013)	100	2,871	IC_50_

For each dataset, the number of tested kinases and compounds is reported, together with the type or provided readout: K_d_ (dissociation constant); K_i_ (inhibition constant); % Activity (remaining catalytic activity); % Control (percentage of kinase bound to the inhibitor compared to a control); IC_50_ (half maximal inhibitory concentration); **Δ**T_m_ (thermal stability shift upon inhibitor binding); *: not all kinase/inhibitor combinations were tested.

Additionally, a large and growing number of known three-dimensional (3D) structures of whole kinases or kinase domains are available in the Protein Data Bank (PDB, Berman et al., 2013), and, in few cases, the kinase was also co-crystallized with an inhibitor. These structures provide a rich background for a detailed analysis of kinase binding pockets and for a better identification of binding determinants.

Computational methods for kinase/inhibitor relationships analysis and inference were successfully attempted in the past (e.g., Manallack et al., 2002; Vieth et al., 2004; Xia et al., 2004; Chuaqui et al., 2005), but were limited by the incomplete and heterogeneous data available at the time. In this review we focused on recent computational methods and resources that employ the latest kinase inhibition profiling data but go beyond standard quantitative structure-activity relationship (QSAR) modeling approaches, which are generally specific for a single target, being instead purposely tailored toward kinase inhibition analysis and applied to the whole kinome, taking advantage from the overall kinase domain conservation and from shared binding patterns and characteristics and providing multidimensional structure-activity relationships concerning tens or hundreds of targets at the same time (Goldstein et al., 2008).

## METHODS FOR KINASE/INHIBITOR INFERENCE

Procedures that use inhibition data from panels of proteins tested against panels of compounds are generally based on numerical descriptions of physicochemical, structural and/or geometrical properties of both ligands and targets, and seek possibly non-linear relationships that explain the binding profiles. Machine learning methods are therefore particularly suited, either for classification (binds/does not bind) or regression on the measured inhibition values (e.g., IC_50_ or K_d_). Since all information available for any kinase target and/or inhibitor is used for learning, these studies can be considered a multi-target approach. Additionally, they can be used to infer novel kinase/inhibitor relationships, also for kinases and compounds not included in the training set.

A number of recent papers explored this kind of approach, differing in the employed training dataset, in the way compounds and proteins are described and in the learning algorithm, but following similar pipelines. For example, Niijima et al. (2012) and Cao et al. (2013) both started from data extracted from Kinase SARfari [in Niijima et al. (2012) the Metz dataset was additionally used for external validation], and propose a similar kinase/inhibitor deconvolution approach, in which the whole kinase sequences, or only the kinase ATP-binding pockets, are deconstructed into residues (either described simply by amino acid type or by physicochemical characteristics) and compounds into chemical fragments or in topological Daylight fingerprints. Yabuuchi et al. (2011) developed a method, called CGBVS (chemical genomics-based virtual screening), in which compounds were represented by a large set of substructure descriptors and physicochemical properties, and protein descriptors were computed from the protein sequence dipeptide composition using a string kernel. Originally developed for G-protein-coupled receptor inhibitors, the method was also applied to kinases, using a panel of 143 kinases and 8830 inhibitors, for a total of more than 15,000 tested interactions extracted from the commercial GVK Biosciences kinase inhibitor database. In Lapins and Wikberg (2010), starting from the Karaman dataset, compounds were described by physicochemical and geometrical characteristics, while kinases were described with either alignment-independent or alignment-based methods, by building a multiple alignment of the kinase domains, excluding gap-rich positions, describing columns of the alignment with physicochemical properties, and applying principal component analysis (PCA) and partial least squares discriminant analysis to summarize descriptors. Schürer and Muskal (2013) employed the Eidogen-Sertanty KKB Q4 2009 release, including more than 430,000 tested kinase/compound pairs extracted from literature and patents. Given the heterogeneous nature of the dataset, data were subject to filtering, standardization, and clustering procedures. For each kinase in the dataset, active and inactive compounds were described using extended connectivity fingerprints, and negative instances for training were either known as inactive on a given kinase, or taken as the entire set of molecules not tested on that kinase.

Then, in these works, machine learning algorithms were trained on kinases and compounds converted into numerical descriptors, to learn associations between kinase residues and compound fragments, and for inference. Variants of a naïve Bayesian (NB) classifier or of a support vector machine (SVM) were used in Niijima et al. (2012), a random forest (RF) in Cao et al. (2013), SVM, decision trees, k-nearest neighbors, and partial least squares projections in Lapins and Wikberg (2010), an SVM in Yabuuchi et al. (2011), Laplacien-corrected NB classifiers, k-nearest neighbors, and partial least squares regression in Schürer and Muskal (2013). All these studies achieved good prediction performances: from 0.67 to 0.73 correlation coefficient in Lapins and Wikberg (2010); accuracy between 74 and 81% and matthews correlation coefficient (MCC) between 0.3 and 0.48 in different tested datasets and with different encodings and learning methods in Niijima et al. (2012); 94% accuracy and 0.98 area under the ROC curve (auROC) in Cao et al. (2013). In Schürer and Muskal (2013), the auROC for individual kinase models vary from around 0.93 to 1, and the prediction accuracy showed a positive correlation with the number of known inhibitors available for training. In Yabuuchi et al. (2011), some predicted novel inhibitors for the epidermal growth factor receptor kinase and the cyclin-dependent kinase 2 were experimentally confirmed, sometimes showing scaffold hopping (i.e., having radically different characteristics than known inhibitors).

Another class of methods includes those taking advantage of kinase 3D structures, used to obtain a more accurate representation of kinase binding sites. A reasonable assumption is that the affinity that a kinase, or a set of kinases, show toward a compound can be ascribed to set of residues that either allow or hinder the binding, and that, once identified in the 3D structures, can be looked for in other kinases to infer their binding ability, even for those kinases for which the 3D structure is unknown, by taking advantage of the kinase domain sequence conservation. Such sets of residues can additionally be converted in numerical descriptors for machine learning.

A subset of kinase/inhibitor pairs extracted from the Fabian and Karaman datasets was used in Caffrey et al. (2008). For these inhibitors the structure of the kinase/compound complex is known, and the specificity determinants can be rationalized. An algorithm was developed to predict specificity determinants given a kinase multiple sequence alignment and structural information, which was able to reproduce the known determinants and to highlight non-trivial additional factors, and can be used as basis for the design of drugs with a desired specificity.

X-React^KIN^ (Brylinski and Skolnick, 2010) is a machine learning method for assessment of cross-reactivity in which each human kinase domain structure was obtained through homology modeling, and binding sites residues were predicted using computational methods. Similarity between kinases was computed by different metrics using sequence, structure, and ligand binding profiles. The system employed data from the Fabian and Karaman panels for training and validation of a NB classifier, obtaining sensitivity higher than 0.5 for around 70% of the tested compounds, and the Bamborough dataset was used for further validation, finding significant correspondence (0.53 average Pearson correlation) between predicted and experimental activity profiles. The computed cross-reactivity profiles are freely available for download.

In Huang et al. (2010), all kinase 3D structures available in the PDB at the time were superposed to obtain a fine description of a series of features known in the literature to be related to inhibitor specificity, e.g., the size of the gatekeeper residue, that affects the pocket accessibility, the hydrogen bonding and covalent bonding ability at specific positions, the flexibility of the hinge loop connecting the kinase domain small and large lobes, and others. These features were extended to kinases for which the structure in unknown via multiple alignments, converted into numerical vectors and used to estimate a similarity between each pair of kinases. Using these distances, a network of kinase binding sites was constructed, which recapitulated well a network based on the similarity between the inhibitor profiles in the Karaman dataset. Integration of the binding site similarity network with the inhibition profile network led to inference of off-target interactions, some of which were validated experimentally.

On the same lines, in Anderson et al. (2012), starting from the Karaman dataset, first kinases were clustered by similarity in binding affinity profiles for the inhibitors tested in the dataset. Kinases within the same cluster were shown to have more similar binding sites, as detected by the comparison of the binding site 3D structures extracted from the PDB. *In silico* docking procedures then highlighted cluster-specific residues acting as interaction hot spots, which were converted into a series of descriptors, used for RF training, achieving 76% of prediction accuracy. The RF was then used for the prediction of novel kinase/inhibitor relationships, some of which were experimentally tested, obtaining a good agreement with the predicted K_i_ values in 70% of the cases.

The Karaman dataset, crossed with kinase 3D structures available in the PDB, were also the starting point for the work presented in Bryant et al. (2013); the structure of a kinase bound to a known type II kinase inhibitor, *imatinib*, was used as template to identify contact residues, mapped to all other considered kinases using the Pfam (Punta et al., 2012) kinase family multiple alignment. A combinatorial clustering was used to find subsets of binding site residues that better correlate with the binding affinities reported in the Karaman dataset. An SVM was then trained on these data, and the prediction performance was estimated individually for each inhibitor as the auROC, which ranges from 0.5 to 1 (mean 0.8). Finally, the trained SVM was used to infer the binding ability of unlabeled kinases.

## INTEGRATIVE APPROACHES

The wealth of kinase inhibition profiling data presents great opportunities for being analyzed as a whole, by integrating data from different resources in order to provide a unified view on kinome inhibition. The whole kinase/inhibitor data can therefore be represented as a network, where binding can be treated as a binary on–off relation or weighted by the binding affinity or by the strength of the inhibitory effect. This kind of network can aid in the identification and rationalization of drugs secondary effects and facilitate drug repositioning.

KIDFamMap (Chiu et al., 2013) and K-Map (Kim et al., 2013) are free web-databases in which kinase/inhibitor relationships, retrieved from different sources, are connected and integrated with other annotations to facilitate the at-a-glance investigation of the kinome inhibition. In KIDFamMap, the Karaman, Anastassiadis and Davis profiling panels, Kinase SARfari, the PDB, and others resources, for a total of more than 186,000 kinase/compound pairs, are investigated by decomposing each interaction into a series of binding pocket sub-regions and compound fragments preferences (Chen et al., 2010), and then extending the identified rules to the whole kinome (introducing the concept of kinase/inhibitor families) and associated to known pathologies involving kinases. Queries can start from a kinase, a compound or a disease, retrieving a detailed overview of the kinase/inhibitor interaction, all the other interactions belonging to the same family, and a description of associated diseases and how allelic variants might affect the compound binding. In K-Map the Anastassiadis and Davis datasets were analyzed by building connectivity maps based on the Kolmogorov–Smirnov statistic to find correlations between inhibitors and lists of kinases. K-Map allows querying these datasets by kinase, kinase family, custom lists, or kinase-related GO terms, obtaining lists of associated inhibitors ranked by correlation significance. Similarly, the user can start from lists of inhibitors. The intent of K-Map is to provide insights for drug development and repositioning.

Caveats of integrative approaches are that to convert data into an on-off relation would require setting thresholds that might not be easy to optimize, and that data from different sources might not be directly comparable, so they must be opportunely processed. In Sutherland et al. (2013) the Anastassiadis, Metz and Davis datasets were compared to each other and to an additional profiling panel (the Sutherland dataset in **Table 1**), by converting each readout in an estimated IC_50_, testing the concordance between IC_50_ in different panels, and for promiscuity and selectivity measures. They found that the all panels have good agreement in assessing whether a compound is active or inactive on a given kinase, but the exact inhibition values show instead low levels of concordance, as well as measures of how much selective is a compound.

In Tang et al. (2014) the Metz, Davis, and Anastassiadis datasets were compared and integrated with data from ChEMBL and STITCH. Since these panels employed different assays and different readouts (K_d_, K_i_ and percentage of remaining activity for the Davis, Metz and Anastassiadis datasets, respectively), a new method called KIBA (kinase inhibitor bioactivity) is introduced to obtain a single comparable activity score for each kinase/compound pair. The three panels have a relatively small number of common tested kinase/inhibitor pairs; in such cases, the Metz and Davis datasets show good degree of correlation between readouts, which is smaller when both are compared with the Anastassiadis panel. The project resulted in a kinase/inhibitor bioactivity map comprising 467 kinases and more than 50,000 compounds, which is freely available.

## CONCLUSION

While different in methods and scope, the approaches presented here highlight the need for original and effective computational methods to unravel the rich and complex kinase/inhibitor relationships systematically measured in inhibition profiling panels, which can have significant implications in understanding the reasons of the inhibition, helping in the rational design of bioactive molecules, and can be used for the *in silico* prediction of inhibition for those neglected kinases for which no systematic analysis has been carried yet, and for the selection of inhibitors with desired promiscuity. Additionally, a better understanding of the kinase determinants of inhibition can help in apprehending the different response of individual patients to treatment, such as inhibitor resistance due to specific mutations, moving toward a more personalized treatment.

## Conflict of Interest Statement

The authors declare that the research was conducted in the absence of any commercial or financial relationships that could be construed as a potential conflict of interest.
